# Phages in a thermoreversible sustained-release formulation targeting *E*. *faecalis in vitro* and *in vivo*

**DOI:** 10.1371/journal.pone.0219599

**Published:** 2019-07-10

**Authors:** Mor Shlezinger, Michael Friedman, Yael Houri-Haddad, Ronen Hazan, Nurit Beyth

**Affiliations:** 1 Department of Prosthodontics, Hebrew University-Hadassah School of Dental Medicine, Jerusalem, Israel; 2 Faculty of Dental Sciences, Hebrew University-Hadassah School of Dental Medicine, Jerusalem, Israel; 3 Department of Pharmaceutics, The Institute for Drug Research, Faculty of Medicine, The Hebrew University, Jerusalem, Israel; University of Kansas, UNITED STATES

## Abstract

**Introduction:**

*Enterococcus faecalis* is a key pathogen recovered from root canals when conventional treatment fails. Phage therapy has generated new interest in combating pathogens. A sustained-release formulation using specific phages against *E*. *faecalis* may offer an alternative approach.

**Objectives:**

To evaluate the efficacy of anti-*E*. *faecalis* phages formulated in a thermo- sustained-release system against *E*. *faecalis in vitro* and *in vivo*.

**Methods:**

EFDG1 and EFLK1 phages were formulated with poloxamer P407. Gelation time, phage survival, activity and toxicity were evaluated. Lytic activity was evaluated *in vitro* against *E*. *faecalis* at various growth phases, including anti-biofilm activity. Methods included viable bacterial count (CFU/mL), biofilm biomass determination and electron microscopy (live/dead staining). Further evaluation included infected incisors in an *in vivo* rat model. Anti*-E*. *faecalis* phage-cocktail suspension and sustained-release phage formulation were evaluated by viable bacterial count (CFU/mL), histology, scanning electron microscopy (SEM) and 16S genome sequencing of the microbiota of the root canal.

**Results:**

Gelation time for clinical use was established. Low toxicity and a high phage survival rate were recorded. Sustained-release phages reduced *E*. *faecalis* in logarithmic (4 logs), stationary (3 logs) and biofilm (4 logs) growth phases. Prolonged anti-biofilm activity of 88% and 95% reduction in biomass and viable counts, respectively, was recorded. Reduction of intracanal viable bacterial counts was observed (99% of enterococci) also seen in SEM. Phage treatment increased *Proteobacteria* and decreased *Firmicutes*. Histology showed reduced periapical inflammation and improved healing following phage treatment.

**Conclusion:**

Poloxamer P407 formulated with phages has an effective and long-lasting effect *in vitro* and *in vivo* targeting *E*. *faecalis*.

## Introduction

*Enterococcus faecalis* (*E*. *faecalis*) is one of the common pathogens recovered from patients suffering from recurrent root canal treatment failures [1]. The ability of *E*. *faecalis* to form biofilm both on the root canal walls and within the dentinal tubules contributes to their persistence [2]. Moreover, the complex structure of the root canal system allows bacterial evasion from the immune system and antibiotics [3].

Currently, endodontic treatment against root canal infections involves biomechanical cleaning of the canal and disinfectant irrigation, followed by sealing and restoration [3]. The use of antimicrobial agents can improve the prognosis of endodontic treatment by reducing the bacterial load [4]. Antiseptic and antibiotic materials are used against intracanal bacterial infection. For example, chlorhexidine is a common effective intracanal irrigant, yet at high concentrations, it may irritate the surrounding tissue [5]. Another example is sodium hypochlorite, which has been shown to be an effective irrigant against *E*. *faecalis* biofilm in dentinal tubules of bovine incisors [6]. Unfortunately, long after the completion of treatment and sealing, *E*. *faecalis* can be detected. This compromises the treatment outcome and results in treatment failure [7] and in persistent inflammation that is called "secondary apical periodontitis". The microbial population of treated roots associated with secondary apical periodontitis consists of gram-positive facultative anaerobes, including *Enterococcus*, *Streptococcus* and *Lactobacillus*. *Enterococcus faecalis* is the most common species [8]. Moreover, *E*. *faecalis* was reported to be found in samples from every type of endodontic infection and was encountered more frequently in treated root canals of teeth evincing post-treatment disease [9].

Bacteriophages (phages) are bacterial viruses that can lyse bacteria. Phage therapy has generated new interest, as it offers an alternative means to deal with unmanageable infections mostly involving multidrug resistant bacteria and biofilms [10, 11]. One of the advantages of phage therapy is the ability to target a specific bacterial strain, thus leaving the normal flora unharmed [12–14].

More than 25 phages against *E*. *faecalis* were isolated and previously described [3]. Most of them belong to the *Myoviridae* or the *Siphoviridae* families of tailed phages [3]. Paisano *et al*. showed that phage therapy may be an important alternative for root canal treatment, demonstrating *in vitro* that a significant reduction of *E*. *faecalis* bacteria could be achieved with phage treatment [13]. Khalifa *et al*. isolated and described the anti-*E*. *faecalis* EFDG1 phage and EFLK1 phage [10, 15]. Specifically, EFDG1 phage was found to be highly effective against *E*. *faecalis* biofilm *in vitro* [10] and against this bacterium in extracted teeth in an *ex vivo* model [10]. Furthermore, the use of a phage cocktail was suggested as a better solution to the emergence of resistant bacteria compared to single phage use [15–17]. The synergistic effect of different phages enables the use of small doses of phages that lead to faster elimination of the bacteria [18]. *In vivo* treatment with anti-*E*. *faecalis* phages EFDG1 and EFLK1 in a phage cocktail rescued 100% of fulminant peritonitis ill mice and did not alter the gut microbiome [19].

It has been shown that the outcome of phage treatment *in vivo* depends on the dose of the phage delivered at the infection site [17]. Unfortunately, the stability of phages in solution is limited because the processing and storage of phages causes a decrease in phage titer [17]. Formulating phages in different materials may help prolong the phage activity [17]. Thus, the chemical and physical properties of the formulation into which the phages are inserted should be considered. The most common methods described to stabilize, immobilize and encapsulate the phages are spray-drying, spray freeze drying, freeze drying, emulsion and polymerization techniques [17]. Other delivery options of phages were described, including chewing gum [20], syrup composition [21] and polycaprolactone/collagen I nanofibers [22]. An example of a successful local delivery of phages was reported in wound healing when dealing with *Staphylococcus aureus* contamination of the skin [23, 24]. Another example is the sustained-release of the T4 bacteriophage, which was found to be effective in food preservation [25].

Poloxamers are nonionic copolymers that were previously evaluated as emulsifying agents for intravenous fat emulsions, solubilizing agents in syrups and as wetting agents for antibacterials [10, 22–27]. The viscosity of poloxamers changes according to the temperature, making them thermoreversible gels [28]. Poloxamers have a low viscosity at 4°C and a high viscosity at 25°C [27]. Sustained-release treatment of chlorhexidine and silver nanoparticles in root canal infection was shown to decrease the intracanal bacterial load [26]. It can be speculated that the thermoreversible properties of poloxamer P407 may be advantageous for root canals. The potential of sustained-release phage treatment against *E*. *faecalis* is unexplored. Herein, we investigate the use of poloxamer P407 as a sustained-release formulation for phages. We hypothesized that phages could target *E*. *faecalis* effectively when given as a sustained-release drug.

## Materials and methods

### Materials

Unless otherwise stated, all materials were purchased from Sigma-Aldrich (St. Louis, MO).

### Bacterial and phage strains

*E*. *faecalis V583* (ATCC 700802) served as the test organism. For each experiment an aliquot of frozen bacteria was thawed and transferred into a test tube containing brain heart infusion (BHI) medium (Difco, Detroit, MI) and grown for 24 h at 37°C under aerobic conditions. Then the cultures were diluted 1:1000 into a new tube with fresh BHI medium and grown 3 h to mid log phase (optical density (OD) = 0.5) for exponential cultures or 24 h for late stationary cultures. In this work, we used EFDG1 and EFLK1 phages that were previously isolated and characterized [10, 15]. EFDG1 and EFLK1 phages were dialyzed to a phosphate-buffered saline (PBS) medium using 10,000 MWCO SnakeSkin Pleated Dialysis Tubing (Thermo scientific, Meridian Rd., Rockford, USA) for 24 h at 25°C mixed with a magnetic stirrer. The PFU/mL of the phages did not change during the dialysis. Khalifa *et al*. reported that the best treatment against *E*. *faecalis* biofilm was achieved by a 1:1 ratio of EFDG1 and EFLK1 in a phage cocktail [15]. Thus, all the experiments in this study were performed using a cocktail with a 1:1 ratio of the two phages.

### Formulation

Kolliphor P 407 (CAS: 9003-11-6, BASF, Ludwigshafen, Germany) poloxamer was added to the previously prepared solution of phage cocktail with 10^9^ PFU/mL EFDG1 and 10^9^ PFU/mL EFLK1 and magnetically stirred at 4°C.

### Assessment of gelation time

The gelation time of the phage-poloxamer P407 formulation was evaluated at different concentrations of the poloxamer: 25%, 28% and 30%. One milliliter of solution at 4°C was pipetted into a glass tube and incubated at 37°C. The solidification time of the solution to gel was recorded. Further investigation utilized only the 30% poloxamer solution (prepared by adding 2.1 g poloxamer to 7 mL phage solution), which showed the shortest solidification time (S1 Table).

### Assessment of the phage release rate from the formulation

Three hundred microliters of phage-poloxamer formulation (poloxamer 30% of total volume) at 4°C were pipetted to a 6.5 mm Transwell with a 5 μm pore polycarbonate membrane insert (Sterile product no. 3421, Costar, Corning Incorporated, NY 14831, USA). Poloxamer formulated with PBS served as a control. Each formulation was examined with 5 repeats. The inserts were incubated at 37°C for 10 minutes for solidification. Then, in a 24-well plate, 1 mL of PBS was added to each well. The PFU/mL of the phages released from the formulations to the PBS was measured using the standard double-layered agar method [15] after 1, 2, 8, 14, 21, and 28 days. After sampling the phages released into the solution, the inserts were moved to new wells, PBS was inserted, and the plate was incubated at 37°C until the next PFU/mL measurements were taken.

### Assessment of lytic activity against *E*. *faecalis*

Twenty microliters of formulated 30% poloxamer and phage cocktail (10^9^ PFU/mL EFDG1 phage and 10^9^ PFU/mL EFLK1 phage) were pipetted on the sidewall of the wells in a vertically standing 96-well microtiter plate (Thermo Fisher Scientific, Cat no. 1067008, Denmark), followed by a 10 minutes incubation at 37°C. *E*. *faecalis V583* logarithmic (10^5^ colony forming units CFU/mL) or stationary (10^9^ CFU/mL) cultures were added to each well, and the lytic activity was assessed. The growth kinetics of the cultures were recorded at 37°C with 5 s of shaking every 20 minutes in a 96-well plate reader (Synergy; BioTek, Winooski, VT) at 650 nm (OD)_._

### Assessment of lytic activity against *E*. *faecalis* biofilm

*E*. *faecalis* biofilms were grown in BHI broth for 1 week in a 24-well microtiter plate at 37°C, as previously described [10]. To produce a one-week biofilm the medium was replaced every 24 h with a fresh medium. One hundred microliters of formulated poloxamer-phage were added to a 6.5 mm Transwell with a 5 μm pore polycarbonate membrane insert at 4°C, and the inserts were transferred to the biofilm plate using a sterile tweezer. Once the experiment began supplementary medium was added to allow bacterial nutrition without biofilm disturbance or without losing any of the sustained release phages present in the medium. Thus, fresh medium was added at a ratio of 1:3 every 48 h ensuring 1 mL medium in each well during the experimental period. The biofilm was treated at 37°C for periods of 1, 3, 7, 14, 21, and 28 days. Viable counts (CFU/mL) were evaluated. Wells were scraped thoroughly, with particular attention given to the well edges. Each of the well contents was transferred to a 1.5 mL tube and placed in a sonication water bath (Bandelin sonopuls HD 2200) for 5 minutes to disrupt the biofilm, 10 μL of serial dilutions of the sample were plated on BHI. Colonies were counted after 24 h at 37°C. A similarly prepared plate was used to evaluate biofilm biomass using crystal violet staining before and after treatment as previously described [15]. Briefly, at the end of the treatment period, the inserts were removed, and the wells were gently washed with PBS to remove unattached bacteria. One milliliter of methanol was added to each well, followed by incubation for 20 minutes. The methanol was then aspirated, and the wells were air-dried. The biofilms were stained with 1 mL of crystal violet (1%) for 20 minutes at 25°C and then washed with water. A 1 mL volume of ethanol was added, and the biomass was determined at 538 nm OD. Further investigation included similarly prepared plates to evaluate the dead/live bacterial ratio in the biofilm following treatment. After the inserts were removed, the wells were washed gently with PBS to remove unattached bacterial cells and then processed with a live/dead cell viability kit (Life Technologies. Waltham, MA). The fluorescence emissions of the samples were detected using a Zeiss LSM 410 confocal laser microscope (Carl Zeiss). Red fluorescence was measured at 630 nm, and green fluorescence was measured at 520 nm. Horizontal plane optical sections were made at 5-μm intervals from the surface outward, and the images were displayed individually. The microscopy slices were combined to form a 3D image using Bioformats and UCSD plugins (ImageJ 1.49G). The stained biofilms were examined using a confocal microscope and analyzed using ImageJ 1.49G software (http://imagej.nih.gov/ij/).

### Toxicity tests

The toxicity was tested on macrophages, since they are the most abundant immune cell found in a periapical lesions in humans [29]. Furthermore, since macrophages holds a large number of pathogen recognizing receptors (since they belong to the antigen presenting cells family), these cells are most likely to react against any foreign body. Macrophages are quick to react, and thus enable the detection of adverse host response to any agent. Sixty thousand RAW 264.7 (ATCC TIB71) macrophage mice cells were incubated for 24 h in a 96-well plate in Dulbecco's Modified Eagle's Medium-high glucose (DMEM) combined with fetal calf serum (Biological Industries, USA). Twenty microliters of 4°C phage-poloxamer formulation was pipetted on the sidewall of the wells in a vertically standing 96-well microtiter plate (Thermo Fisher Scientific, Cat no. 1067008, Denmark), followed by a 10 minutes incubation at 37°C for solidification. Fresh media was added to each well. Cells activated by 10 μL *Porphyromonas gingivalis* ATCC 33277 that were heat killed by a 10 minutes incubation at 80°C served as a positive control as was previously described [30]. The viability of the cells was evaluated using a colorimetric XTT assay (Cell Proliferation Kit (XTT based) Biological Industries, USA), as described by Scudiero *et al*. [31]. The assay is based on the ability of metabolically active cells to reduce the tetrazolium salt XTT to orange colored compounds of formazan. In brief, following 24 h incubation at 37°C, 50 μL of XTT labeling mixture were added to each well and the microplates were incubated for a further 4 h. A Vmax microplate reader (Molecular Devices Corporation) with a 450 nm optical filter and a 650 nm reference wavelength was used to measure the absorbance of each well.

### Assessment of phage lytic activity *in vivo* in a rat root canal infection model

**Fig 1:**
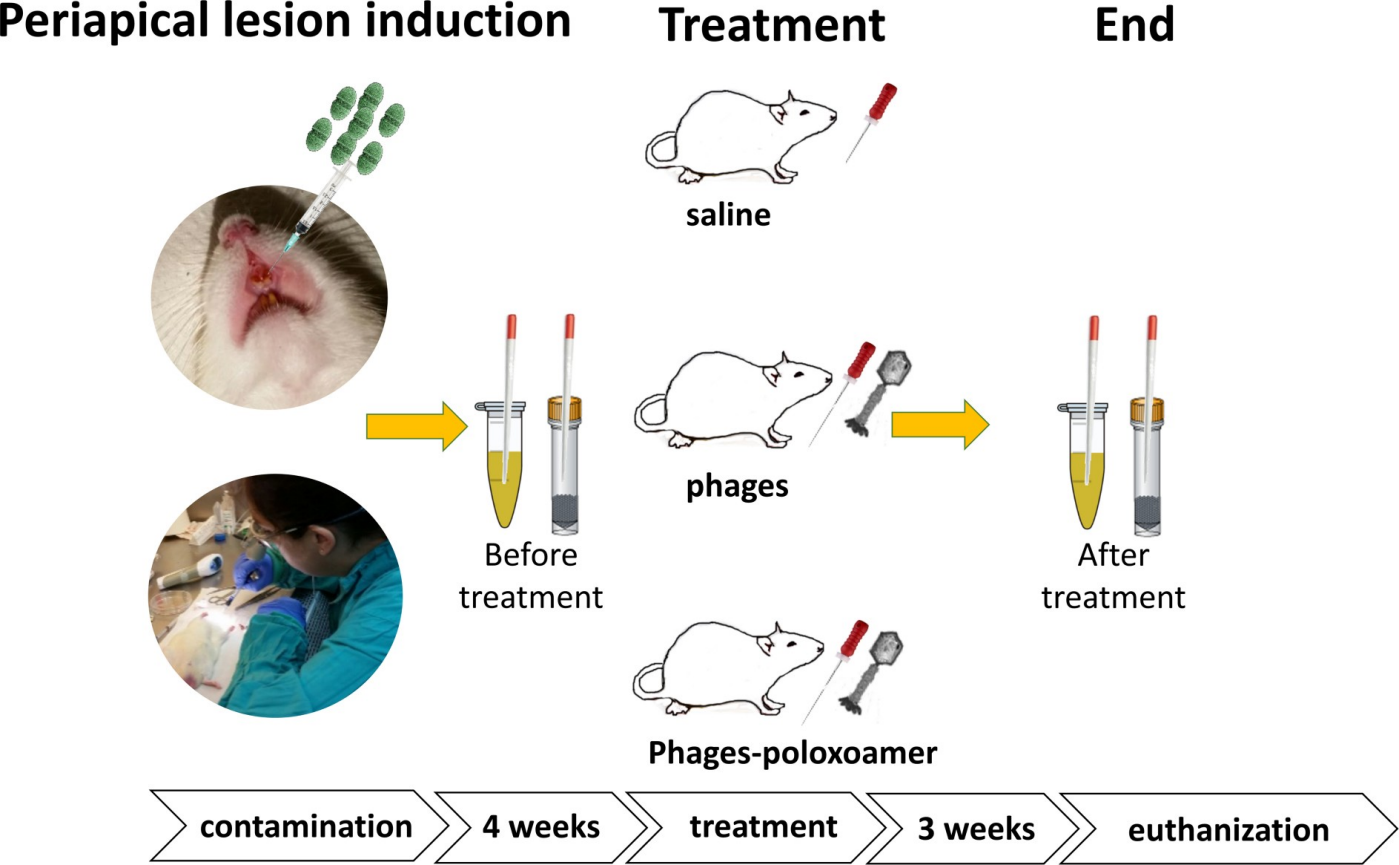
Periapical lesions were induced in a rat model. Dental pulp of the maxillary incisor teeth of male Wistar rats were exposed and infected with *E*. *faecalis* (VRE ATCC 700802). Standard root canal treatment was conducted using instrumentation and one of the following treatments: group A: saline irrigation; group B: EFDG1/EFLK1 phage cocktail irrigation (10^9^ PFU/mL); group C: EFDG1/EFLK1 poloxamer-phage formulation (10^9^ PFU/mL).

All procedures that involved animals were approved by the Hebrew University Ethics committee (Ethics number: MD16-14688-4). Evaluation included 64 maxillary incisor teeth of 32 male Wistar rats (250–300 g body weight, 12 weeks old, 8 animals per group). At day 1, rats were anesthetized by an intraperitoneal (IP) injection of ketamine 1 g/10 mL (Vetoquinol, Lure, France) at 40–80 mg/kg body weight combined with xylazine 20 mg/mL (Eurovet Animal Health, B. V. Bladel, Netherlands) at 5–10 mg/kg body weight. Intra muscular injection of tramadol HCl 100 mg/2 mL (Grunenthal, Aachen, Germany) at 5 mg/kg was given for pain relief. Pulp exposure was performed on maxillary incisor teeth by cutting the teeth 2 mm coronally to the gum using surgical scissors (The Medical Export Group B.V., The Netherlands) followed by cutting with a carbide round bur size ¼ (Meisinger, Germany) to 1 mm depth. Endodontic files #10 and #15 K (Micro-Mega, Besancon, France) were used to mechanically prepare the root canal. Fifty microliters of *E*. *faecalis* (1×10^9^ CFU/mL) was injected into each root canal using a 1 mL syringe (BD Plastipak, Spain) (Fig 1). Exposed pulps were left open to the oral cavity to enhance the formation of periapical lesions for 30 days [32]. *E*. *faecalis* (1×10^9^ CFU/mL) in 2% carboxymethyl cellulose (CMC) was given orally to the rats at days 7 and 14. At day 30, rats were anesthetized by an IP injection of ketamine 1 g/10 mL, 40–80 mg/kg body weight combined with xylazine 20 mg/mL, 5–10 mg/kg body weight. Root canals were performed with a #25 k file, and the canals were irrigated as follows: group 1—saline irrigation; group 2—EFDG1/EFLK1 phage cocktail; and group 3—EFDG1/EFLK1 phage cocktail-poloxamer formulation. Phage-poloxamer formulation was kept at 4°C until application and was injected into the root canals with a syringe followed by intracanal spreading with 25 mm lentulo spiral paste fillers (Henry Schein, Switzerland) during the gelation process. The root canals were sealed using single bond universal adhesive (3M, Neuss, Germany) and Filtek Ultimate Flowable Restorative (3M ESPE, St. Paul, USA). Microbiological samples were collected by #25 sterilized paper points from the maxillary incisors and were inserted to sterile PBS before and 3 weeks after treatment. Ten μL of serial dilutions of the sample were plated on Enterococcosel agar (Becton, Le Pont de Claix, France) plates with aerobic incubation for evaluating intra-canal *enterococci* viable bacterial number (CFU/ mL). Bacterial DNA from the root canal was evaluated by sampling the root canals with #25 sterilized paper points that were inserted to TD1 solution (UltraClean Tissue & Cells DNA Isolation Kit, MO BIO, Carlsbad, CA). Three weeks after treatment, periapical lesions were evaluated using scanning electron microscopy and histology. In addition, the changes in the microbiota of the root canals were evaluated using 16S metagenomic sequencing.

### DNA isolation

The bacterial DNA from the root canals was sampled using sterilized paper points followed by isolation using UltraClean Tissue & Cells DNA Isolation Kit Sample Catalog No. 12334-S (for Genomic DNA), according to the manufacturer's protocol. The DNA was stored at ˗20°C.

### 16S metagenomic sequencing library preparation

The 16S ribosomal RNA gene amplicons were prepared for the Illumina MiSeq System according to the manufacturer’s protocol as was previously described [19]. Briefly, libraries were prepared using two PCR steps. In the first PCR step, the V3 and V4 regions of the 16S rRNA gene (460 bp) were amplified using 25 cycles with 5 μL of the extracted DNA. The full-length primer sequences, using standard IUPAC nucleotide nomenclature, to follow the Illumina protocol targeting this region were: 16S Amplicon PCR Forward Primer = 5′ TCGTCGGCAGCGTCAGATGTGTATAAGAGACAGCCTACGGGNGGCWGCAG and the Reverse Primer = 5′ GTCTCGTGGGCTCGGAGATGTGTATAAGAGACAGGACTACHVGGGTATCTAATCC. The PCR products were visualized using TapeStation System (Agilent, Santa Clara, CA, USA) with D1000 ScreenTape System (Agilent, Santa Clara, CA, USA). The PCR products were purified using AMPure XP beads (Beckman Coulter, CA, USA). In the second PCR step Nextera XT Index kit (Ilumina) primers were used to label the first PCR product using 8 cycles. The second PCR products were purified using AMPure XP beads. All purified PCR products were pooled together and normalized to 10 nM. The 10 nM stock was diluted to 4 nM, which was denaturated using NaOH and 20% PhiX control. The libraries (10pM) were then sequenced in the Illumina Miseq (Ilumina, San Diego, CA, USA) using the V2 kit for 500 cycles (250x2, paired end reads).

### Diversity-related statistical tests

The statistical tests for the intracanal bacterial DNA were carried out in R Studio using the vegan and phyloseq packages. Alpha diversity was assessed by the Shannon diversity index.

### Scanning electron microscopy

The maxilla of the rats was sagittally cut between the incisors and was fixed in Karnovsky’s fixative (2% PFA, 2.5% glutaraldehyde in 0.1 M cacodylate buffer, pH 7.4) for 4 h at 20°C, followed by a 50% dilution with Karnovsky’s fixative (diluted in 0.1 M Cacodylate buffer) and overnight incubation at 4°C. The samples were then postfixed in 1% OsO_4_ in 0.1 M cacodylate buffer for 2 h, dehydrated through a graded alcohol series, and placed in a critical point dryer (Quorum Technologies, K850 Critical Point Drier Ashford, Kent, England). After sputtering (Quorum Technologies, SC7620 Spatter coater) with Au/Pd, samples were viewed under SEM (FEI, Quanta 200, Czech Republic).

### Histology

The maxilla of the rats was sagittally cut between the incisors and was fixed in Karnovsky’s fixative (2% PFA, 2.5% glutaraldehyde in 0.1 M cacodylate buffer, pH 7.4), followed by addition of 0.1 M Cacodylate buffer 48 h later. The samples were then prepared by decalcifying solution (50% formic acid, 20% sodium citrate) for 7 days. The sagittal slicing was performed in 3 semi-serial sections of 5 μm thickness slices every 200 μm and stained with hematoxylin & eosin. Periapical tissues were histomorphometrically analyzed for signs of inflammation and periapical bone resorption. The soft tissue widening (including periodontal ligament, connective tissue, inflammatory infiltrate) and bone resorption depicting inflammation were quantified using the Periapical Index (PAI) as previously described [33, 34]. Briefly, categories include: 1- mild chronic inflammation, 2—moderate chronic inflammation, 3—severe chronic inflammation, 4 -severe inflammation with features of exacerbation.

### Statistical analysis

The results were analyzed as the mean ± standard error of 8 animals in each experimental group. Statistical significance was calculated by Student’s t-test two tailed unpaired (significance level: p < 0.05).

## Results

### Gelation time

Gelation time was assessed to determine the poloxamer concentration resulting in the shortest solidification time. The gelation time was 2 minutes and 20 seconds with poloxamer at 30% of the total phage solution volume. Poloxamer concentrations of 25% and 28% of the total volume were solidified after 3 minutes and 10 seconds (S1 Table). The poloxamer concentration of 30% of the total solution volume was chosen and used in all following experiments.

### Phages were released from poloxamer P407 after 1 month

**Fig 2:**
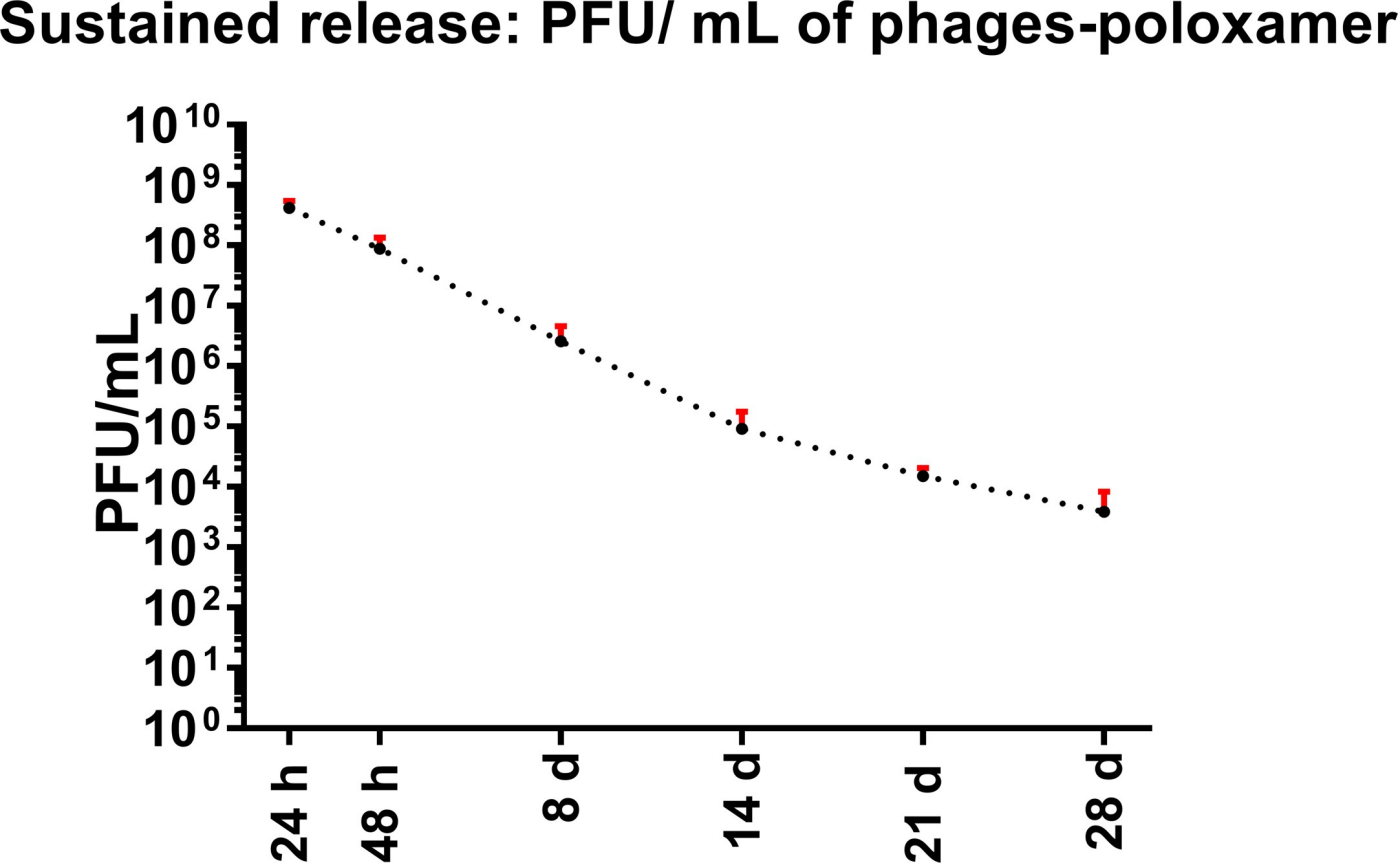
Poloxamer-phage cocktail releases active anti-*E*. *faecalis* phages for a month. PFU/mL of the EFDG1/EFLK1 phage cocktail released from 30% poloxamer-phage formulation measured after 1, 2, 8, 14, 21 and 28 days. Phage release was measured for a month. A high concentration of phage release was observed during the test period, and the phage concentration of the solution gradually decreased from 10^9^ to 10^3^ PFU/mL. The results are based on 5 independent biological replicates.

Formulation of phages in poloxamer P407 medium did not hinder phage survival when used at 37°C, as shown in Fig 2. This survival was evident for a month. Constant release of phages was recorded, demonstrating a gradual decrease in the phage concentration (PFU/mL) over 1 month. Release of EFDG1 and EFLK1 phages after formulation with poloxamer P407 was evident after 1, 2, 8, 14, 21, and 28 days, and the phage cocktail showed antibacterial activity against *E*. *faecalis*. The phages were highly effective against *E*. *faecalis* after 24 h (4×10^8^ PFU/mL) compared to a control sample of PBS-poloxamer that did not affect the bacteria. After 28 days, phages were still released from the poloxamer with an infectivity of 3.8×10^3^ PFU/mL (Fig 2).

### Poloxamer-phage formulation kills planktonic *E*. *faecalis*

**Fig 3:**
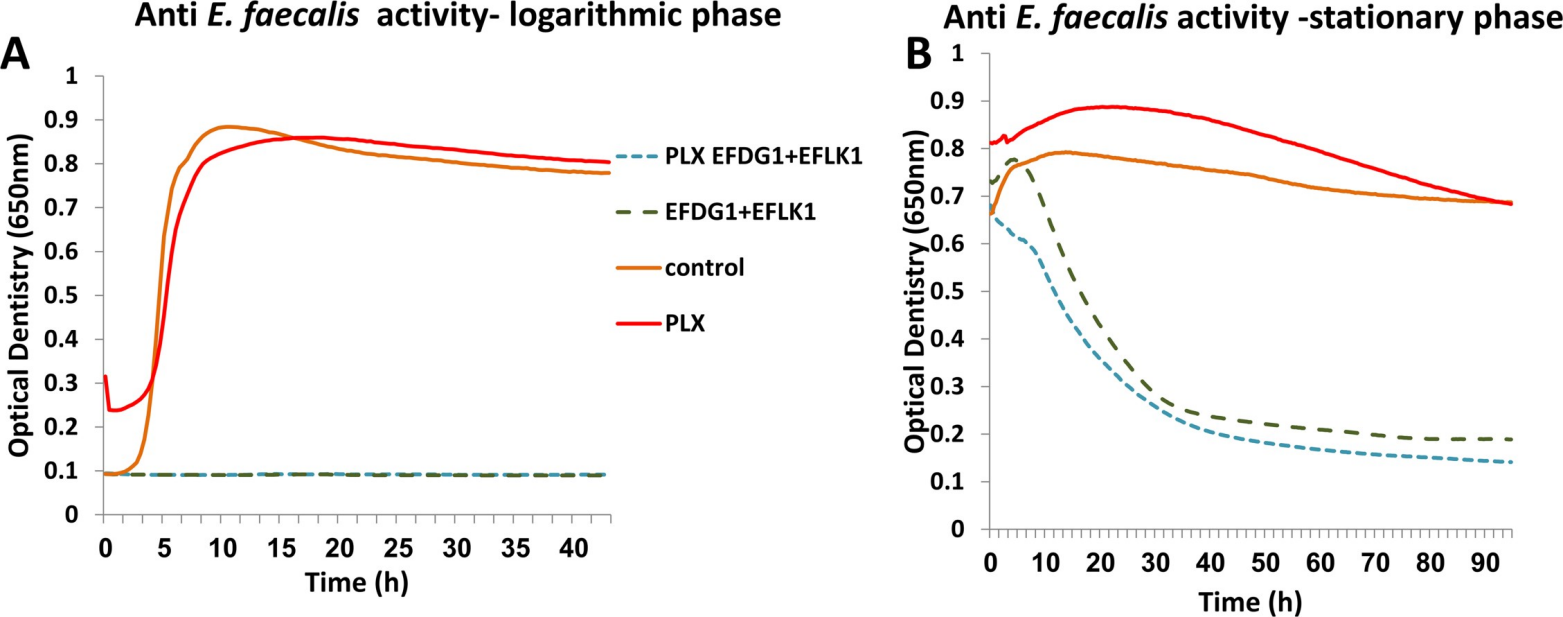
Poloxamer-phage formulation kills logarithmic and stationary *E*. *faecalis*. **(A) Treatment of bacteria in the logarithmic growth phase with phages and poloxamer-phage formulation reduced bacterial growth.** Treatment with phages and a poloxamer-phage formulation (dashed curves: EFDG1/EFLK1 phage cocktail) reduced bacterial growth of logarithmic *E*. *faecalis* compared with the control (untreated bacteria and poloxamer alone), as depicted by OD changes. The results are based on 6 independent biological replicates.**(B) Treatment of *E*. *faecalis* in the stationary growth phase with phages and poloxamer-phage formulation reduced bacterial growth.** Treatment with phages and a poloxamer-phage formulation (dashed curves: EFDG1/EFLK1 phage cocktail) reduced bacterial growth of stationary *E*. *faecalis* compared with that of cells in stationary phase treated with poloxamer alone (PBS based) and untreated bacterial control as depicted by OD changes. The results are based on 6 independent biological replicates.

Phages released from the poloxamer-phage formulation killed bacteria in the logarithmic phase efficiently, as demonstrated by the reduction in the OD (Fig 3A). Poloxamer formulated with PBS did not affect bacterial growth. The same effect was evident against stationary phase bacteria (Fig 3B).

### Poloxamer-phage formulation targets *E*. *faecalis* biofilm

**Fig 4:**
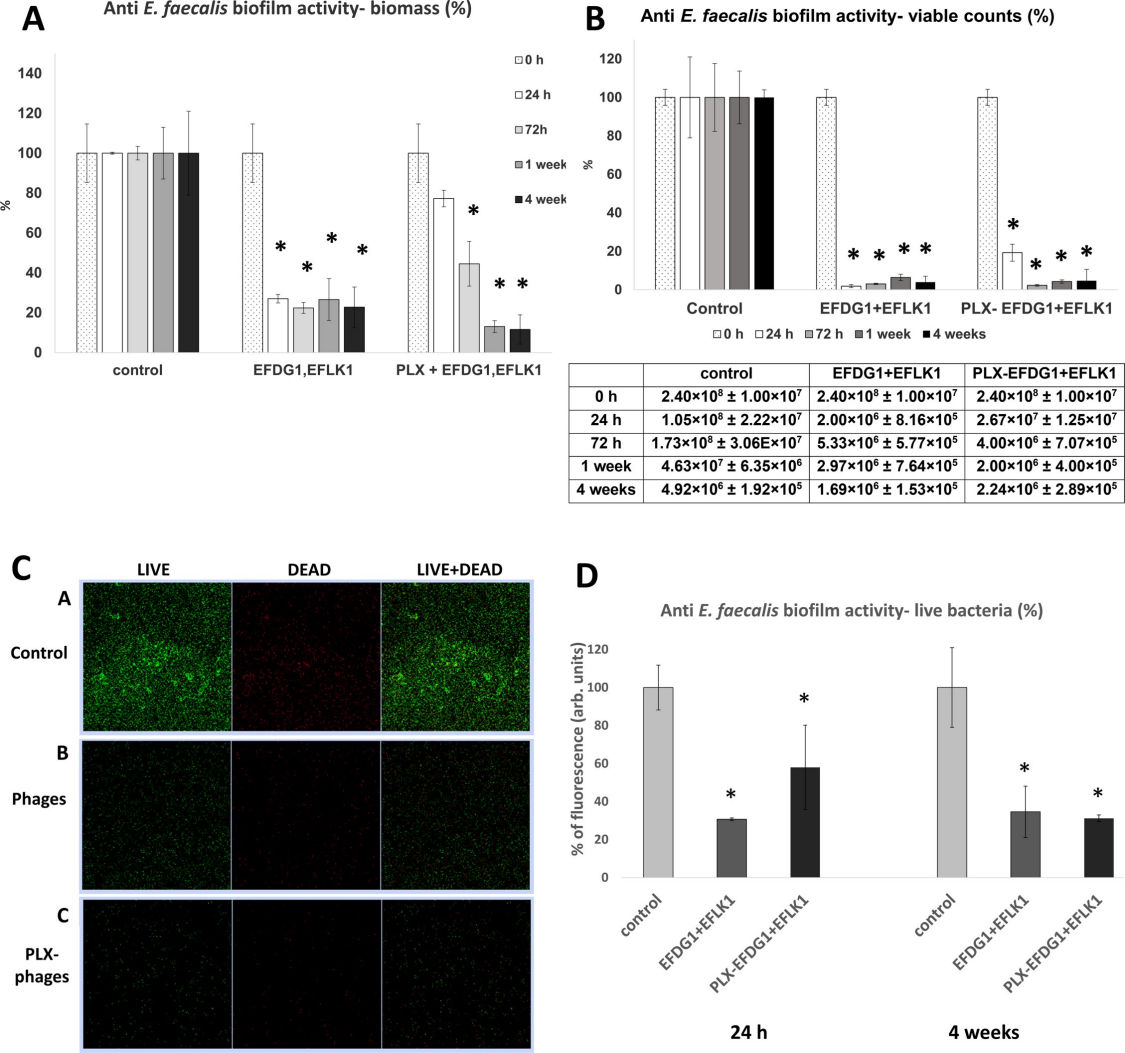
Sustained-release of phages targets *E*. *faecalis* biofilm. **(A) *E*. *faecalis* biofilm biomass decreased following treatment with sustained-release EFDG1/EFLK1 phage cocktail.** Treatment with phages and a poloxamer-phage formulation decreased bacterial biofilm biomass, as evaluated by crystal violet staining after 0 h (dots), 24 h (white), 72 h (light gray), 1 week (dark gray), and 4 weeks (black). After 24 h, the phage suspension showed the best antibacterial activity, but after 4 weeks of treatment, the poloxamer-phage formulation reduced biofilm mass most efficiently. The results are presented as percentages, normalized to the biofilm biomass controls. Statistical significance was calculated by Student’s t-test (significance level: p < 0.05) compared to the untreated control. The results are based on 8 independent biological replicates. **(B) *E*. *faecalis* biofilm viable bacterial counts decreased following treatment.** The viable counts of *E*. *faecalis* biofilm bacteria (CFU/ mL) after 0 h (dots), 24 h (white), 72 h (light gray), 1 week (dark gray) and 4 weeks (black) after treatment with phage suspension and poloxamer-phage formulation are described. Treatment with phage suspension reduced the viable bacterial counts by more than 90% during all treatment periods. Treatment with poloxamer-phage formulation reduced the bacterial counts by more than 90% after 72 h of treatment. The results are presented as percentages, normalized to the viable counts controls. The CFU/mL values of the viable counts and the standard deviation are detailed in the table below. Statistical significance was calculated by Student’s t-test (significance level: p < 0.05) compared to the untreated control. The results are based on 8 independent biological replicates. **(C) Treatment with EFDG1/EFLK1 poloxamer-phage formulation and EFDG1/EFLK1 phage suspension targeted *E*. *faecalis* after 4 weeks.** Confocal microscopy of live/dead cells in stained biofilm: [A] *E*. *faecalis* untreated biofilm shows bacterial clusters. [B] EFDG1/EFLK1 phage suspension-treated *E*. *faecalis* biofilm shows lysis of the bacteria. [C] EFDG1/EFLK1 poloxamer-phage formulation-treated *E*. *faecalis* biofilm shows lysis of the bacteria. **(D) Decrease in green-stained cells of *E*. *faecalis* biofilm following treatment with EFDG1/EFLK1 poloxamer-phage formulation and EFDG1/EFLK1 phage suspension.** Treatment with phage suspension and poloxamer-phage formulation decreased green stained bacteria, as depicted by live/dead cell staining after 24 h and 4 weeks visualized by confocal microscopy (Zeiss). The results are presented as percentages, normalized to the untreated controls. Statistical significance was calculated by Student’s t-test (significance level: p < 0.05) compared to the untreated control. The results are based on 8 independent biological replicates.

The sustained-release of phages into biofilm was more effective than application of the phage suspension, as observed after 4 weeks, inducing an 88% (SD ± 7%) reduction in the bacterial biofilm biomass compared to the phage suspension, which reduced the biofilm biomass by 77% (SD ± 10%) (Fig 4A). After one week of treatment, the sustained-release activity showed a 86% (SD ± 3%) decrease compared with the phage suspension, which reduced the biofilm biomass by 73% (SD ± 10%) (Fig 4A). The *E*. *faecalis* biofilm viable bacterial count showed that the sustained-release activity of poloxamer-phage formulation (PLX-phage) was as effective as the phage suspension after 72 h, reducing the CFU/mL by 97% (SD ± 0.4%) compared to the untreated biofilm control (Fig 4B). The antibiofilm effect was observed for a month (Fig 4B). Accordingly, confocal microscopic images depicted 70% (SD ± 0.65%) and 65% (SD ± 13%) decreases in green-stained cells after 24 h and four weeks, respectively, following treatment with the phage suspension. The antibacterial activity of the poloxamer-phase formulation was more efficient after 4 weeks, showing a 68% (SD ± 1.7%) decrease in green-stained cells compared to a 42% (SD ± 22%) decrease after 24 h (Fig 4C and 4D).

### Poloxamer-phage formulation is not toxic to cells

**Fig 5:**
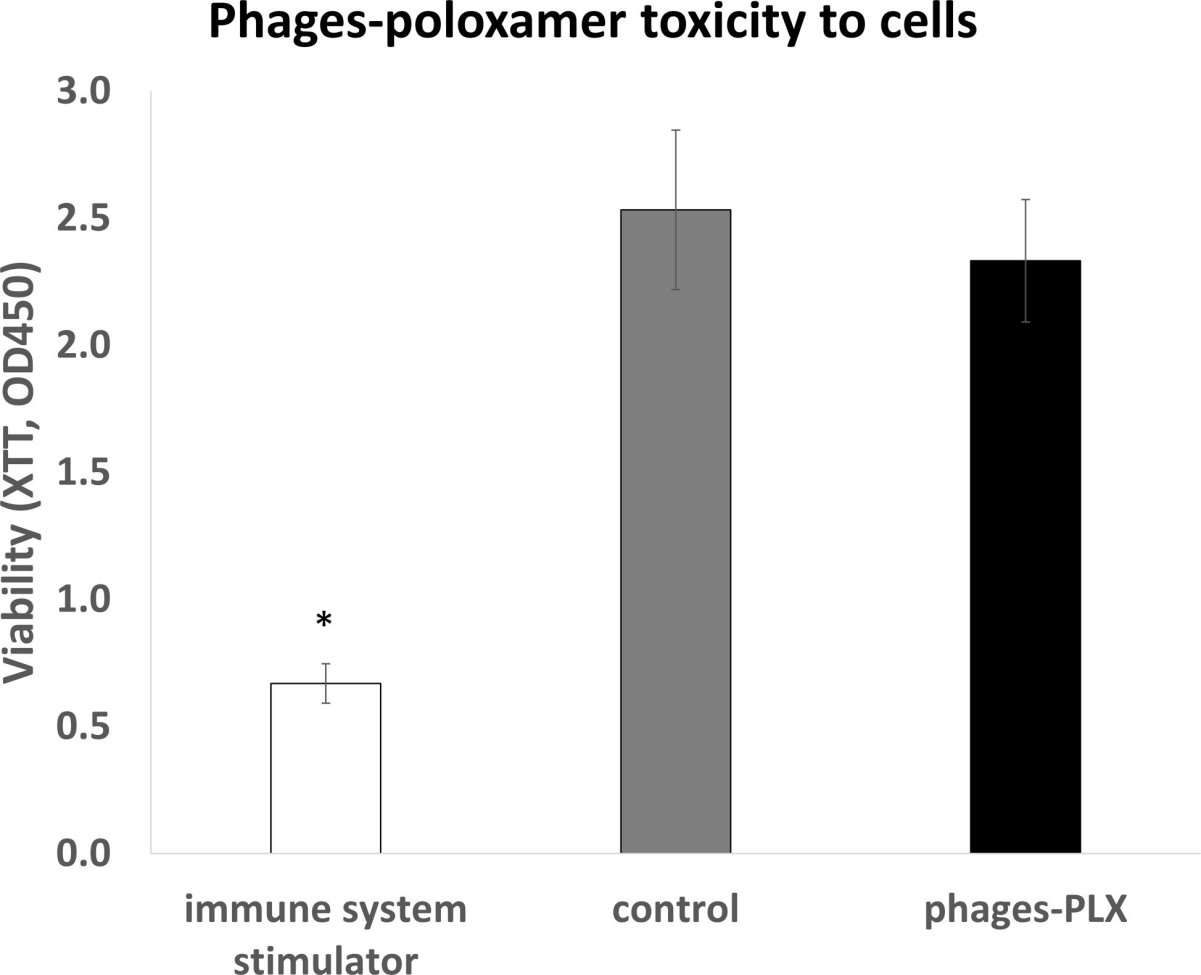
Poloxamer-phage formulation is not toxic to cells. XTT viability indicator shows that RAW macrophage cell viability was unaffected by poloxamer-phage formulation treatment compared to an immune system stimulator (heat killed *Pg*). The decrease in the OD_450_ of the tested group was insignificant (p > 0.05). White = immune system stimulator, gray = untreated control, black = phages-poloxamer. Statistical significance was calculated by Student’s t-test (significance level: p < 0.05) compared to the untreated control. The results are based on 8 independent biological replicates.

RAW macrophage mouse cell viability was unaffected by the poloxamer-phage formulation treatment, demonstrating similar viability rates (p = 0.17) to the untreated macrophages (Fig 5).

### *In vivo* treatment with poloxamer-phage formulation showed a decrease in *E*. *faecalis* infection of root canals in rats

**Fig 6:**
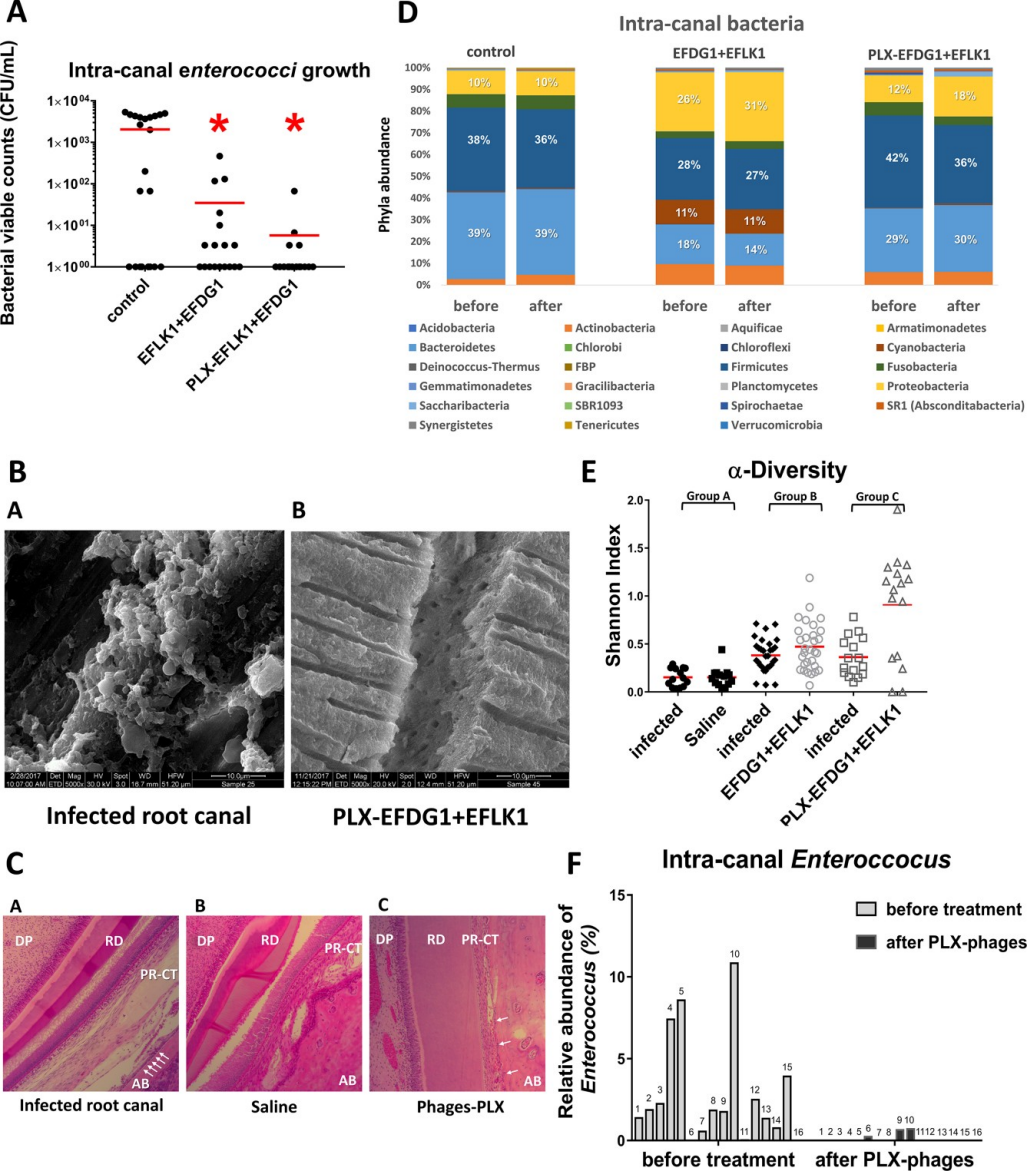
Phage treatment against VRE periapical infection in rats. **(A) Intracanal viable enterococci counts after treatment with EFDG1/EFLK1 phage suspension and EFDG1/EFLK1 poloxamer-phage formulation.** Viable counts of enterococci show a significant decrease following phage treatment. Control = saline irrigation, EFLK1+EFDG1 = EFDG1/EFLK1 phage suspension irrigation (10^9^ PFU/mL), PLX-EFLK1+EFDG1 = EFDG1/EFLK1 poloxamer-phage formulation (10^9^ PFU/mL). Statistical significance was calculated by Student’s t-test (significance level: p < 0.05) compared to the untreated control (n = 14–16 teeth in every tested group). **(B) Scanning electron microscopy: no survival of bacteria in rat dentinal tubules.** (magnification: X5000) [A] control: *E*. *faecalis*-infected tooth shows biofilm inside the dentinal tubules; [B] *E*. *faecalis*-infected tooth treated by poloxamer-phage formulation does not exhibit bacteria inside the dentinal tubules. **(C) Histology of *E*. *faecalis-*infected rat incisors followed by root canal treatment with poloxamer-phage formulation showing reduced inflammation.** Histology of sagittal cross-sections of the periapical area of Wistar rat incisors that were decalcified, and H&E stained. [A] *E*. *faecalis-*infected teeth show enlarged peri-radicular connective tissue and osteoclasts suggesting inflammation, bone resorption and apical periodontitis. [B] *E*. *faecalis-*infected teeth that were irrigated with saline show enlarged highly cellular peri-radicular connective tissue and necrosis. Osteoclasts are present in the alveolar bone. [C] *E*. *faecalis-*infected tooth that was treated with poloxamer-phage formulation shows highly vascularized dental pulp, reduced peri-radicular connective tissue, and a woven bone with osteoclast presence. White arrows indicate infiltration of osteoclasts. DP = dental pulp. RD = root dentin. PR-CT = peri-radicular connective tissue. AB = alveolar bone (magnification: × 10). **(D) Changes of the relative abundance of intracanal phyla due to root canal treatments.** The changes in the intracanal microbiota were evaluated using 16S genome sequencing. Intracanal samples were collected 4 weeks after *E*. *faecalis* infection (before treatment) and 3 weeks after root canal treatment. Root canal treatment included instrumentation and treatment with one of the following: group A: Saline irrigation; group B: EFDG1/EFLK1 phage cocktail irrigation (10^9^ PFU/mL); or group C: EFDG1/EFLK1 poloxamer-phage formulation (10^9^ PFU/mL). The most abundant phyla were *Firmicutes*, *Bacteroidetes* and *Proteobacteria*. Phage cocktail suspension and poloxamer-phage formulation treatments caused a decrease in the relative abundance of *Firmicutes* and an increase in the relative abundance of *Proteobacteria*. (n = 14–16). **(E) Alpha diversity dot plot representing taxa richness changes before and after root canal treatment (Shannon index) and differences between the treatment groups.** Standard root canal treatment with instrumentation and group A: saline irrigation; group B: EFDG1/EFLK1 phage cocktail irrigation (10^9^ PFU/mL); or group C: EFDG1/EFLK1 poloxamer-phage formulation (10^9^ PFU/mL). Higher values of alpha diversity (via Shannon index) were obtained following poloxamer-phage formulation and phage cocktail suspension treatment compared to the other groups. **(F) Relative enterococcal abundance changes before and after poloxamer-phage formulation treatment.** Representative data from 16S genome sequencing at the genus level reveals an enterococcal decrease following poloxamer-phage formulation treatment. 16 incisor teeth are presented in each group before and after treatment. Gray = 4 weeks after *E*. *faecalis* infection. Black = 3 weeks after poloxamer-phage formulation treatment (7 weeks after infection).

Enterococci bacterial growth in the intracanal 3 weeks after root canal treatment in rats showed a decrease in viable bacterial counts (CFU/mL) after treatment with the phage cocktail, with better antibacterial activity achieved after use of the sustained-release phage formulation; Viable enterococci count showed a 99% decrease following treatment with the poloxamer-phage formulation (SD ± 0.81%), compared with 95% (SD ± 5.03%) following treatment with the phage cocktail suspension (Fig 6A).

### Phage treatment reduced the bacterial load inside the dentinal tubules

The antibacterial effect was depicted by SEM micrographs of sagittally cut maxillary incisor root dentin from rats. A reduction in the bacterial load in the dentinal tubules was depicted following all treatment modalities compared to the untreated control. Treatment with sustained-release phage cocktail eliminated most of the bacteria inside the dentinal tubules (Fig 6B).

### Reduced periapical inflammation following poloxamer-phage treatment

The histology of dental pulp and peri-radicular tissue of *E*. *faecalis-*infected Wistar rat incisors was investigated. Histological analysis of the antibacterial activity on host inflammation in the periapical area showed that PAI score was the lowest after poloxamer-phage formulation treatment (1.85 ± 0.34), compared to the infected root canals and the saline irrigation that were scored 3.71 ± 0.45 and 3 ± 0.75 respectively (Table 1). Saline-treated teeth showed wider peri-radicular tissue, highly cellular dental pulp and osteoclast presence compared to those treated with the poloxamer-phage formulation (Fig 6C).

**Table 1:** Histological analysis of the antibacterial activity on host inflammation in the periapical area—Periapical Index (PAI*) (n = 7 each group).

	PAI 1	PAI 2	PAI 3	PAI 4	PAI average ± SD
**Infected root canal**	0	0	2	5	3.71 ± 0.45
**Saline**	0	2	3	2	3 ± 0.75
**Phages-PLX**	1	6	0	0	1.85 ± 0.34

* Periapical index (Orstavik and Brynolf index [33, 34])

1 = mild chronic inflammation

2 = moderate chronic inflammation

3 = severe chronic inflammation

4 = severe inflammation with features of exacerbation

### A change in the intracanal microbiome followed phage cocktail and poloxamer-phage treatment

A total of 5,753 different operational taxonomic units (OTUs) were identified. The most abundant phyla were *Firmicutes*, *Bacteroidetes* and *Proteobacteria*. Phage cocktail suspension and poloxamer-phage formulation treatment caused a decrease in the relative abundance of *Firmicutes* (6% and 1% decrease in the poloxamer-phage formulation and phage cocktail suspension, respectively) and an increase in the relative abundance of *Proteobacteria* (6% and 5% increase in the poloxamer-phage formulation and phage cocktail suspension, respectively) (Fig 6D). Greater taxa richness represented by higher values of alpha diversity (via Shannon index) was obtained following poloxamer-phage formulation and phage cocktail suspension treatment (Fig 6E). At the genus level, an enterococcal decrease was depicted following poloxamer-phage formulation treatment. The average relative abundance of enterococci before treatment was 2.8% (SD ± 3.26%) compared with 0.098% (SD ± 0.23%) following poloxamer-phage formulation treatment (p = 0.002) (Fig 6F).

## Discussion

Intra-canal treatment using sustained-release phage formulations holds promise in *E*. *faecalis* bacterial load reduction, where conventional treatment fails. It has been shown that long after the root canal treatment is completed and sealed, *E*. *faecalis* is detected, inducing treatment failure [7]. Herein, we show that phages not only kill planktonic *E*. *faecalis*, but they effectively target and eliminate *E*. *faecalis* biofilm *in vitro* as well as *in vivo* inside the dentinal tubules and the root canal walls when used via the new delivery method including a poloxamer-phage formulation. The poloxamer-phage formulation can be easily used in daily clinical life, allowing prolonged and highly effective anti-*E*. *faecalis* effect with no evidence of toxicity to animal cells or to animals.

Poloxamer P407 was chosen to serve as the sustained-release matrix for the phages due to several advantages related to this material, especially its thermoreversible properties, which allow it to exist as a liquid at 4°C and a gel at 25°C [27]. The low viscosity allows for intracanal injection of the material and solidification after it is spread thoroughly throughout the cavity. It takes two minutes and twenty seconds for the poloxamer-phage formulation to turn to gel (S1 Table) at 25°C, a relatively normal working time compared to other dental materials, such as calcium hydroxide, which sets in 2.5 to 5.5 minutes [35]. This system may be appropriate for daily clinic use due to its working and setting times. Formulating phages with the poloxamer matrix did not decrease the antibacterial activity of the phages (10^9^ PFU/mL, MOI 1 = 1), that was evident for a month (Fig 2). The prolonged antibacterial activity of the phages and the gel consistency of the matrix make poloxamer-phage formulations an ideal intracanal medicament material. Serving as an antimicrobial agent that is placed in the root canals between endodontic treatment appointments it will prevent reinfection by remaining microorganisms [36]. Calcium hydroxide, the most commonly used intracanal medicament with antibacterial properties that is based on hydroxide ion release [37], showed better results after 7–14 days of intracanal dressing compared to a single endodontic treatment appointment without dressing of the root canal [38, 39]. At least two weeks are needed to create bactericidal activity by calcium hydroxide in the periapical area [40], yet calcium hydroxide-based sealers are effective against most of the microorganisms in the root canals [41], with little or no effect on *E*. *faecalis* [8, 42]. In this study, we suggest an equivalent antibacterial system that uses sustained-release phages to eliminate the intracanal bacterial load. Both phage formulations and calcium hydroxide-based materials have similar setting times, prolonged antibacterial activity and are easy to use. The main advantage of the suggested phage treatment is that it is highly effective against *E*. *faecalis*, unlike calcium hydroxide. Phages released from the poloxamer as well as phages in suspension decreased logarithmic and stationary *E*. *faecalis* v583 optical density dramatically (Fig 3).

Finding an effective anti-*E*. *faecalis* biofilm treatment is essential to overcoming the problem of recurrent root canal treatment failures, as *E*. *faecalis* is one of the common pathogens in these cases. Bacterial cells that have been organized in a biofilm matrix are known to be inaccessible to antibacterial agents and to the immune system [43], contributing to the chronicity of these infections [44]. The persistence of *E*. *faecalis* in root canals is increased due to its ability to form biofilms both on the root canal walls and within the dentinal tubules [2]. Defeating biofilm infection is a challenging task [43]; therefore, it takes more time and requires longer antibacterial activity, making a sustained-release treatment extremely relevant. Formulating phages in the sustained-release matrix of poloxamer showed an effective antibacterial activity after a week of treatment, reducing the bacterial biomass by 88% and the viable bacterial counts by 95% (Fig 4). The highly effective antibacterial activity against biofilm lasted for a month, an important advantage of the poloxamer-phage formulation when considering it as an intracanal medicament. Interestingly, after 24 h the phage suspension (10^9^ PFU/mL) was more effective against the biofilm compared to the sustained release phages (4×10^8^ PFU/mL at 24 h), possibly due to easier penetration of non-formulated phages into the complex structure of the biofilm.

Choosing phages for *in vivo* treatment needs to be done carefully by verifying [43]: that they do not carry harmful genes in their genome sequence; that they are effective against every life stage of the bacteria (Figs 3 and 4) [15] and that neither the phages nor the poloxamer have toxicity towards animal cells (Fig 5). Before using phages as a therapy in humans, it is crucial to determine, in an animal model, that there is no immune response [45], that the phages keep their stability and that there are no problems in the delivery of the treatment. We created an *in vivo* model (Fig 1) that best resembled human root canal infection and apical periodontitis in which we examined intracanal treatments. The initial sample after four weeks confirmed an infection of the canal. Unlike previous *in vivo* studies of phage treatments against *E*. *faecalis* that considered a systemic treatment [19, 43], treating apical periodontitis requires a local treatment that will be able to navigate the maze of the dentinal tubules to the bacterial infection. Root canal treatment in live Wistar rats that included a mechanical preparation of the root canal, phage medicament and coronal sealing of the teeth was performed. The treatment showed a decrease in the bacterial load and a specific decrease in the viable cell count of enterococci. A previously described sustained-release treatment for root canal infection decreased the intracanal bacterial load [26]. In our study, the poloxamer-phage treatment showed a better antibacterial effect than the phage cocktail suspension (Fig 6). We speculate that this is due to several reasons: first, because of the long-lasting antibacterial properties of the poloxamer-phage formulation. Additionally, the gel consistency of the poloxamer-phage formulation maintained the material inside the canal, unlike the washable phage suspension. No systemic reaction to the local phage treatment occurred in the rats. Interestingly, the peri-radicular connective tissue was wider in the teeth that were not treated by the poloxamer-phage formulation suggesting apical periodontitis because of a local response to the infection (Table 1 and Fig 6C). Chronic apical periodontitis is a result of a localized inflammatory response that induces apical bone resorption caused by osteoclasts [46]. The presence of a woven bone and osteoclasts together with the narrow PDL width suggests a healing process of the peri-radicular tissue in the poloxamer-phage-treated teeth (Fig 6C).

We found that in the local environment of root canals, the irrigation and medicament materials altered the microbiome composition. At the phylum level, phage treatment provoked an increase in the relative abundance of *Proteobacteria*. Previously, *Proteobacteria* were found to be the most abundant phylum in root canals associated with persistent apical periodontitis [47]. Consistent with our findings, it was previously described that in the case of sodium hypochlorite and chlorhexidine-treated apical periodontitis, the most prevalent phyla were *Firmicutes*, *Fusobacteria*, *Bacteroidetes* and *Actinobacteria* (Fig 6D and 6E) [48]. Together with changes in the phylum relative abundance, it was interesting to see that phage treatment induced a decrease in enterococci at the genus level (Fig 6F). The microbiome change amounts to a specific change in the target bacteria of the phages [49] and a more general impact on the root canal niche.

Poloxamer-phage formulation effectively targeted every growth stage of *E*. *faecalis in vitro*, including the extremely difficult to treat biofilm. *In vivo* poloxamer-phage treatment showed effective antibacterial activity against an intracanal enterococci infection. Further *in vivo* studies should test phage treatment in molar teeth, which, unlike the rat incisors, have a closed apex. In future investigations, it will be interesting to study a combined treatment of poloxamer-phage and calcium hydroxide. Combining poloxamer-phage, a specific highly effective anti*-E*. *faecalis* treatment with calcium hydroxide, which has a wide antimicrobial range but lacks antibacterial activity against *E*. *faecalis*, may have complementary activity in root canals. The sustained-release properties of the poloxamer-phage formulation are an important advantage that can make the shift between disease and health in root canal treatment and reduce the risk of recontamination by persistent *E*. *faecalis* bacteria.

## Supporting information

S1 Table Poloxamer-phage formulation gelation time. The gelation time of the poloxamer-phage formulation was tested by heating one milliliter of each poloxamer concentration tested from 4°C to 37°C and recording the solidification time of the solution to gel. The 30% poloxamer yielded the shortest gelation time; therefore, it was further investigated.
(DOCX)
