# Supplementary material for: Phages in a thermoreversible sustained-release formulation targeting E. faecalis in vitro and in vivo
Source: PLoS One. 2019 Jul 10;14(7):e0219599. doi: 10.1371/journal.pone.0219599 (PMC6620107; doi:10.1371/journal.pone.0219599)
Supplement: S1 Table — The gelation time of the poloxamer-phage formulation was tested by heating one milliliter of each poloxamer concentration tested from 4°C to 37°C and recording the solidification time of the solution to gel. The 30% poloxamer yielded the shortest gelation time; therefore, it was further investigated. (DOCX) [file pone.0219599.s001.docx]

**Table S1. Phages-poloxamer gelation time.**

| **Gelation time (min)** | **Concentrations of Phages-Poloxamer  (% of total volume)** |
| --- | --- |
| 3'10'' | 25% |
| 3'10'' | 28% |
| 2'20'' | 30% |

The gelation time of phages-poloxamer formulation was tested by transferring one milliliter of each poloxamer concentration tested from 4°C to 37°C and recording the solidification time of the solution to gel. The 30% poloxamer yielded the shortest gelation time; therefore, it was further investigated.
